# Comprehensive Lithium Polysulfide Diffusion Insights within Solid-State Electrolytes

**DOI:** 10.1021/acselectrochem.5c00547

**Published:** 2026-03-25

**Authors:** Marcela de Paula Ramos, Oier Pajuelo-Corral, Nicolas Goujon, Didier Devaux, Irune Villaluenga

**Affiliations:** 1 POLYMAT, Applied Chemistry Department, Faculty of Chemistry, University of the Basque Country UPV/EHU, Donostia - San Sebastián 20018, Spain; 2 Applied Chemistry Department, Faculty of Chemistry, University of the Basque Country UPV/EHU, Donostia - San Sebastián 20018, Spain; 3 IKERBASQUE, Basque Foundation for Science, Bilbao 48011, Spain; 4 LEPMI, Univ. Grenoble Alpes, Univ. Savoie Mont Blanc, CNRS, Grenoble INP, Grenoble 38000, France

## Abstract

Lithium–sulfur (Li–S) batteries are highly attractive for next-generation energy storage applications due to their low cost, high theoretical energy density, and environmental benefits (nontoxicity, reducing reliance on rare metals, etc.). However, the implementation of Li–S batteries in the current battery manufacturing lines is hindered by the polysulfide dissolution and migration from cathode to anode through the liquid electrolyte, leading to irreversible capacity fading, poor Coulombic efficiency, and therefore limited battery lifetime. Replacing liquid electrolytes with solid-state electrolytes is the most promising approach to overcome the polysulfide migration challenge in lithium–sulfur (Li–S) batteries. Nevertheless, a fundamental understanding of the mechanisms governing polysulfide diffusion and the development of effective mitigation strategies are crucial. This perspective aims to provide a detailed overview of polysulfide diffusion mechanisms in solid-state electrolytes based on inorganic, polymer, and hybrid materials for Li–S battery applications. Examples of the challenges associated with polysulfide migration and the mitigation strategies employed in each solid electrolyte are provided. These strategies are focused on protective coatings, chemical modifications, advanced sintering techniques, material design, and computational modeling. Moreover, the relevance of advanced characterization techniques (such as XAS and XPS) to elucidate the complex polysulfide dissolution mechanisms occurring in solid electrolytes is highlighted. The insights presented here provide a critical foundation for future research development of efficient and high-performance solid-state Li–S battery devices.

## Graphical abstract

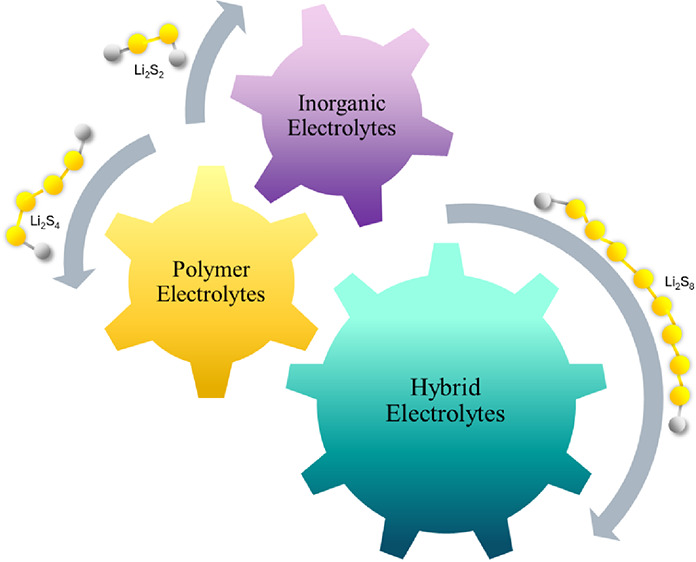

## 1 Introduction

Lithium–sulfur (Li–S) batteries have garnered significant attention as one of the most promising candidates for next-generation energy storage systems, primarily due to their exceptionally high theoretical energy density of about 2500 Wh kg^–1^, which far surpasses that of conventional lithium-ion batteries.^1^ In addition, the use of sulfur as the cathode (i.e., positive electrode) active material offers advantages such as low cost, natural abundance, and environmental benignity, making Li–S batteries an attractive option for applications ranging from portable electronics to electric vehicles and grid-scale energy storage.^2^ However, the practical realization of Li–S batteries is severely impeded by the ″polysulfide shuttle effect”, a phenomenon where the soluble lithium polysulfides (Li_2_S_n_, with 4 ≤ *n* ≤ 8), formed during the electrochemical reaction of sulfur, dissolve in the electrolyte and migrate between the cathode and the anode (i.e., negative electrode) during repeated charge–discharge cycles.^3,4^ This process leads to a cascade of detrimental effects, including the loss of active material, irreversible capacity fading, poor Coulombic efficiency, and thus limited battery lifespan. To overcome these challenges, a fundamental understanding of the mechanisms governing polysulfide diffusion and the development of effective mitigation strategies is essential.^5,6^ In this work, the term “diffusion” refers specifically to the transport of lithium polysulfide species through the electrolyte driven by concentration gradients, representing a mass transport phenomenon intrinsic to the electrolyte properties. In contrast, the “shuttle effect” describes the parasitic electrochemical process in which dissolved lithium polysulfides migrate to the lithium anode, undergo reduction, and subsequently diffuse back to the cathode.

The mitigation of polysulfide shuttle in Li–S batteries is a multifaceted challenge that requires a combination of material design, interfacial engineering, and electrochemical optimization. The strategies can be broadly categorized into five key approaches: (i) physical confinement, (ii) chemical adsorption, (iii) electrochemical kinetic control, (iv) separator modification, and (v) electrolyte optimization.^7–11^ Each approach targets different aspects of polysulfide dissolution, diffusion, and redox reactions, often synergistically combined to achieve superior performance.

Physical confinement strategies aim to trap polysulfides within the cathode or electrolyte structure, preventing their migration to the anode. This is typically achieved through the use of porous materials, core-shell structures, or 3D architectures that create physical barriers.^12–14^ Chemical adsorption strategies rely on the strong affinity between polysulfides and functional materials, often involving polar surfaces or heterogeneous catalysts that promote polysulfide immobilization and conversion.^15^ Controlling the redox reaction kinetics of polysulfides can mitigate their dissolution and diffusion by promoting faster conversion to insoluble Li_2_S_2_/Li_2_S.^16^ The separator plays as well an important role in preventing polysulfide crossover from the cathode to the anode.^17^ Modifying the separator with functional coatings or interlayers can effectively block the polysulfide migration. The electrolyte composition and properties significantly influence polysulfide solubility and diffusion.^18^ Optimizing the electrolyte can suppress the shuttle effect and enhance the battery performance. This last strategy is of high interest as over the past decades, the design and optimization of solid-state electrolytes, which can physically and chemically confine polysulfides while maintaining high ionic conductivity and mechanical integrity, showed very promising results.^19,20^

This perspective provides a comprehensive review of polysulfide diffusion mechanisms in all-solid-state electrolytes for Li–S batteries with a focus on the role of inorganic, polymer, and hybrid electrolytes. We discuss the challenges associated with polysulfide migration, including the degradation of solid electrolytes and the formation of resistive byproducts as well as the strategies employed to mitigate these issues. These strategies include the use of protective coatings, chemical modifications, advanced sintering techniques, and the incorporation of alternative polymers or functional fillers. By synthesizing recent advancements in material design, characterization techniques, and computational modeling, we aim to highlight the potential of solid electrolytes to enable high-performance, long-cycle-life Li–S batteries. The insights presented here are critical for guiding future research toward the development of robust, efficient, and safe solid-state Li–S battery systems that can meet the demands of next-generation energy storage applications.

## 2 Polysulfide Diffusion in Inorganic Electrolytes

Inorganic solid electrolytes are considered to be leading the design of next-generation lithium–sulfur batteries, given their high potential to inhibit the shuttle effect. Inorganic electrolytes are classified into three different types, depending on their chemical nature: sulfide-based electrolytes, oxides, and halides. Despite their solid nature blocking polysulfide migration, each category exhibits distinct behavior. Sulfide solid electrolytes are characterized by a notable intrinsic thermodynamic instability when they interact with sulfur and lithium polysulfide species. Within composite cathodes, the degradation process is aggravated by the presence of electronically conductive materials at the cathode-electrolyte interface.^21^ These conductive components facilitate redox-mediated oxidation and decomposition reactions of the sulfide electrolyte, prompting the formation of secondary reaction products such as Li_2_S and Li_3_P.^22^ The accumulation of these products, which exhibit both electronic and ionic resistance, contributes to the growth of interphase layers that increase the interfacial impedance. This phenomenon not only consumes active sulfur species but also facilitates the transport of polysulfides across the interface. Ultimately, these combined effects lead to capacity fading and the shuttle effect, which undermines the overall performance and longevity of the battery.

Raman and X-ray photoelectron spectroscopy (XPS) are powerful techniques that are usually used to evaluate the degradation of sulfide electrolytes and understand the processes that govern in the electrolytes during battery operation. For example, Lim et al. discovered that Li_6_PS_5_Cl is initially decomposed to Li_3_PS_4_ and finally to P_2_S_*x*_ and S_*x*_ species by Raman spectroscopy.^23^ Similarly, Wenzel *et al.* found the formation of a solid interphase between Li_10_GeP_2_S_12_ electrolyte and lithium metal anode using *in situ* X-ray photoelectron spectroscopy, which revealed the formation of lithium sulfide, lithium phosphide phases, and a Ge–Li and germanium metal alloy, causing an increase of 250 Ω in the resistance after 24 h.^24^ Along with the formation of resistive side products, the degradation of sulfide electrolytes leads to cracks that allow polysulfides to penetrate within the grain boundaries of solid electrolytes. Specifically, the presence of residual porosity and a high grain-boundary area fosters the diffusion of polysulfides, which contributes to the loss of active material and raises internal resistance.^25^

X-ray absorption spectroscopy (XAS) techniques can provide valuable information to study and identify the different polysulfide species formed during battery operation to elucidate the complex reaction pathways occurring during cell cycles.^26^ Each polysulfide (Li_2_S_8_, Li_2_S_6_, Li_2_S···) possesses a characteristic K-edge signature, allowing the identification and quantification of the different sulfur species. Understanding the role of intermediate polysulfides can help develop strategies to avoid unwanted reactions. A simulated sulfur K-edge X-ray absorption spectroscopy database prepared by Guo et al. is available for researchers, allowing the identification of different sulfur species found in lithium–sulfur batteries.^27^ However, the low polysulfide mobility across solid electrolytes makes them difficult to detect at early stages, given their low concentration in the solid electrolyte. Furthermore, it can be challenging when using sulfide solid electrolytes containing sulfur atoms, producing overlapping S K-edge features, which require deconvolution of the obtained signals and the use of computational methods to effectively detect and quantify the different sulfur species. Coupling different techniques can enhance the precision of the extracted data, for example, the combination of X-ray absorption and emission techniques (XESs). In the study of Ishikawa et al., with the combination of XAS and XES, the electronic states and band gaps of sulfur were studied using S K-edge XAS and S Kβ XES. During charging and discharging, peaks for sulfur, lithium polysulfides, and Li_2_S were noted. The bandgaps of the sulfur-active material were narrower than those of sulfur and Li_2_S, suggesting that polysulfides can serve as electronic conduction paths at the sulfur–solid electrolyte interface.^28^ Another complementary technique to XAS could be X-ray photoelectron spectroscopy, since both can probe different aspects of the chemical structure. XPS is ideal for identifying decomposition products at electrode–electrolyte boundaries, where chemical reactions are initiated, whereas XAS can detect chemical changes occurring within the bulk of the solid electrolyte. XPS can study the origin of the degradation at the surface of the electrolyte, and XAS can reveal how the degradation propagates in the bulk of the solid material (Figure 1).^29,30^

**Figure 1:**
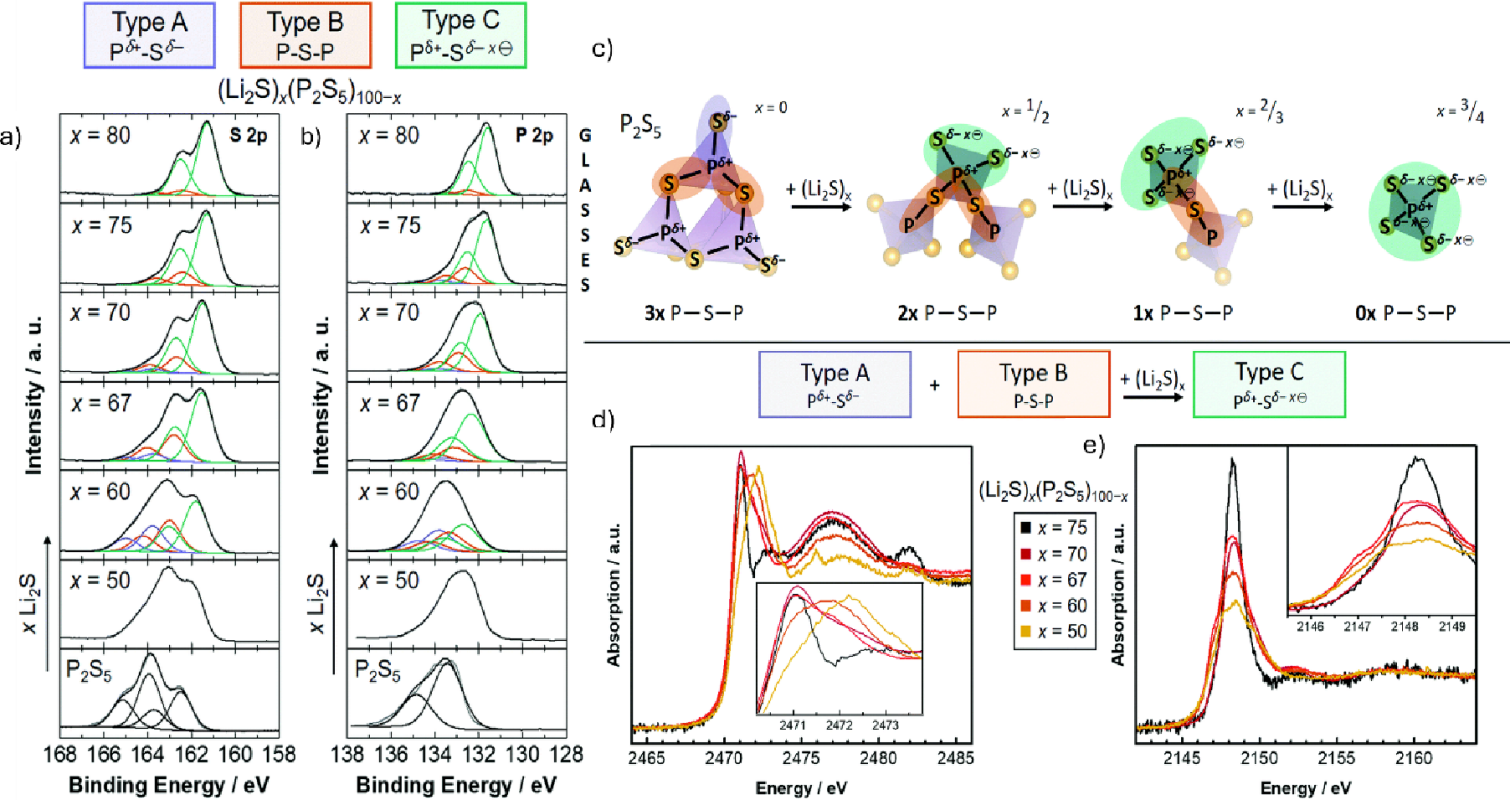
XPS spectra of the sulfur (a) and phosphorus (b) L-edge of LPS glasses and P_2_S_5_. (c) Reaction scheme for P_2_S_5_ upon the addition of Li_2_S. XAS spectra of LPS glasses at the sulfur K-edge (d) and the phosphorus K-edge (e), respectively. Adapted from ref 29.

However, most of the abovementioned techniques to follow polysulfide formation and diffusion are performed *ex situ*, meaning that the different electrochemical reactions occurring during the charge/discharge processes remain unexplored. *Operando* studies are critical to providing real-time information on the electrochemical process during charge–discharge processes in Li–S batteries. Nonetheless, the complex design of the cells makes challenging *operando* measurements, making it easier to perform *ex situ* measurements.^31^ The combination of *operando* and *ex situ* studies can provide an accurate overview of the functioning of the cells during the cycles. For instance, Cao *et al.* effectively combined operando Raman studies with *ex situ* X-ray absorption spectroscopy techniques to deeply study the redox reactions between S_8_ and Li_2_S.^32^ From Raman, the group was able to track the evolution and formation of different sulfur species during real-time cycling, whereas by *ex situ* X-ray absorption, they characterized the lithium–sulfur compounds formed at different charge/discharge stages. The study revealed that sulfur-based batteries with solid inorganic electrolytes cannot only produce polysulfides, but also, they observed the formation of an intermediate metastable Li_2_S_2_ compound, which suggests incomplete reaction and hence reduces the battery’s electrochemical performance. The study performed by Kim *et al.* also supports the formation of Li_2_S_2_ during the discharge process confirmed by XAS and time-of-flight secondary ion mass spectrometry.^33^ The identification of Li_2_S_2_ as an intermediate product is key to picture the global map to understand the origin of the decrease in performance upon different cycles.

Main research lines to prevent polysulfide formation and diffusion in sulfide-based inorganic solid electrolytes are focused on protecting the materials from sulfur cathodes by the application of a layer that acts as a barrier that physically and chemically blocks the migration of polysulfides. For instance, the incorporation of LiF-rich interphases presents an advantageous approach, given the exceptional chemical stability of these interphases, which can serve to provide effective electronic insulation while also facilitating favorable ionic conductivity.^34^ However, concerning environmental and safety considerations, the use of fluorine-based compounds must be avoided or minimized, seeking alternatives that can offer similar benefits without associated drawbacks.^35^ In this sense, the use of halide-based coatings such as LiI, LiBr, and LiCl,^36,37^ has shown similar performances, increasing the chemical stability of the solid inorganic electrolytes while preserving the ionic conductivities and can be easily included by slurry coatings.^38^

Apart from halides, oxide layers between the cathode and the solid electrolyte can successfully block polysulfide migration. Materials such as Li_3_PO_4_, Al_2_O_3_, or LiNbO_3_ are frequently employed for the protection of the electrolytes.^39–41^ Other classes of materials have also been explored for protecting the solid inorganic electrolytes. Yao *et al.* developed a graphene oxide (GO)-coated Li_10_GeP_2_S_12_ sulfide-based solid electrolyte with improved electrochemical performance and stability. Protecting the LGPS sulfide electrolyte with GO is enough to protect the sulfide electrolyte, leading to an improvement in electrochemical performance and excellent cycling stability, with a slight increase in resistance from 38.2 to 78 Ω in the first 100 cycles, and with a remarkable discharge capacity of 830 mA g^–1^ after 750 cycles, demonstrating the long-term cycling stability.^42^

In a similar way, direct contact of sulfides with oxide cathodes leads to parasitic reactions with oxidizable elements, generating an inactive interfacial layer based on SO_*x*_^*n*‑^ and affecting the electrochemical performance.^43,44^ To protect the battery components from degradation, the cathode surface modifications results are attractive. For instance, the group of Ma et al. demonstrated a surface modification of LiNiCoMnO cathode active material (NMC) to effectively prevent LPSCl from degradation.^44^ They proved that when an interfacial layer based on succinonitrile and lithium *bis*(trifluoromethanesulfonyl) imide was applied, no S–O or P–O bonds were observed in high-resolution XPS after long-term cycling, indicating the absence of undesired SO_*x*_^*n*‑^ without compromising the electrochemical performance maintaining a high specific capacity of 168 mAh g^–1^ at 0.1 C and retaining 80% of the capacity after 100 cycles.

Other strategies to increase the stability toward sulfides involve the modification and composition of the sulfide-based electrolyte. For instance, the substitution of sulfur anions with halogens such as iodine or chloride not only enhances the resistivity toward polysulfides, but also increases the electrochemical stability window, improving the overall performance of the Li–S batteries.^45^ The group of Rajagopal *et al.* successfully doped the argyrodite-type solid electrolyte with chlorine anions, improving the conductivity from 5 to 17 mS cm^–1^ in Li_5.3_PS_4.3_Cl_1.7_, and with an electrochemical stability up to 5 V.^46^

In summary, sulfide-based inorganic solid electrolytes are promising candidates for the development of Li–S batteries, derived from their excellent ionic conductivity, softness of the material, and ease of processing. However, despite polysulfide diffusion not being a major problem, they can suffer from degradation in the presence of sulfur species. A multifaceted approach that combines the use of protective coatings and the chemical modification of solid electrolytes is necessary to address the challenges posed by polysulfides, meaning that advancements in material synthesis and protecting methodologies and strategies are key to providing new pathways for the incorporation into Li–S solid batteries.

The well-known oxide solid electrolytes include Li_7_La_3_Zr_2_O_12_ (LLZO), NASICON-type Li_1+*x*_Al_*x*_Ti_2–*x*_(PO_4_)_3_ (LATP), and Li_1.5_Al_0.5_Ge_1.5_(PO_4_)_2_ (LAGP), which are chemically more robust compared to sulfides and exhibit excellent electrochemical stability windows. They have also shown incredibly high electrochemical stability windows and are stable at high voltages (up to 5 V), enabling high-energy densities.^47^ Their lower reactivity with polysulfides and their dense structure hinder the shuttle effect of polysulfides,^48^ making them attractive candidates for Li–S solid batteries. Nonetheless, oxides with higher ionic conductivities are desired, which is the main drawback of their use in Li–S cells. Another attractive feature of this class of materials is their chemical stability and their inert nature, not only toward polysulfides but also toward water and oxygen, all of which simplify the manufacturing process in dry rooms. However, recent studies indicate that even though they do not interact with sulfur atoms, after several cycles, polysulfide diffusion is observed, mainly through the grain boundaries of oxides.^49^

Investigating the diffusion of polysulfides in oxide solid electrolytes presents a significant challenge, primarily due to the inherent chemical reactivity of polysulfides combined with the dense, electronically insulating characteristics of oxide phases. A powerful approach to investigate polysulfide migration in oxide solid electrolytes is the combined use of XPS and time-of flight secondary ion mass spectrometry (TOF-SIMS). Each technique provides complementary information: TOF-SIMS offers high-resolution depth profiles and 2D/3D maps of ionic and molecular species,^50^ while XPS provides chemical speciation, distinguishing between elemental sulfur, polysulfides, Li_2_S, or metal–sulfur bonds. For example, the group of Huang et al. combined both techniques to study polysulfide diffusion in LLTO/polysulfide Li–S cell.^51^ TOF-SIMS depth profiles demonstrated that sulfur signals decline sharply into the bulk electrolyte, indicating restricted diffusion, while XPS confirmed that sulfur remained primarily as elemental S or polysulfides with minimal decomposition products, indicating a stable interface that blocks polysulfide migration. This combined approach offered critical insights into polysulfide diffusion that can provide valuable information to understand and design materials resistant to polysulfide migration.

Looking for increasing the stability of oxides toward polysulfides, the main research lines are focused on microstructural studies to obtain denser oxide electrolytes with pore-free microstructures. Advanced sintering techniques, such as spark plasma^52^ or microwave techniques,^53^ enable the fabrication of highly dense oxide pellets with reduced grain-boundary areas and eliminated residual porosity. In addition, densification of the separator, for example, by hot-pressing, minimizes the available pathways for polysulfides to penetrate, thus enhancing the electrolyte’s blocking capability. The use of sintering additives is also considered a promising route to reduce residual porosity.^54^ As an example, Liu *et al.* decreased the grain-boundary impedance from 1243 to 248.2 Ω, by the addition of 75Li_2_O-12.5B_2_O_3_-12.5SiO_2_ during the sintering process to obtain densified pellets, which, apart from diminishing potential polysulfide diffusion pathways, leads to an enhancement of the total ionic conductivity.^55^

In terms of chemical stability, despite having proven to be stable against sulfur species, significant challenges remain to be addressed regarding long-term stability. While the hardness of metal oxides has demonstrated substantial resistance to polysulfides in the bulk, they are susceptible to suffering degradation after multiple charge–discharge cycles. The group of Naguib *et al.* studied the interfacial stability between LLZO solid electrolyte and a sulfur cathode. Through deep XPS (Figure 2), they found that there is a superficial decomposition at both open circuit and cycled LLZO, being deeper for the cycled sample and which reaction layer can extend to 90 nm depth. This degradation can cause an increase of the resistance from 1400 to 2800 Ω cm^2^ and a capacity decrease from 1154 to 604 mAh g^–1^after 50 cycles.^56^

**Figure 2:**
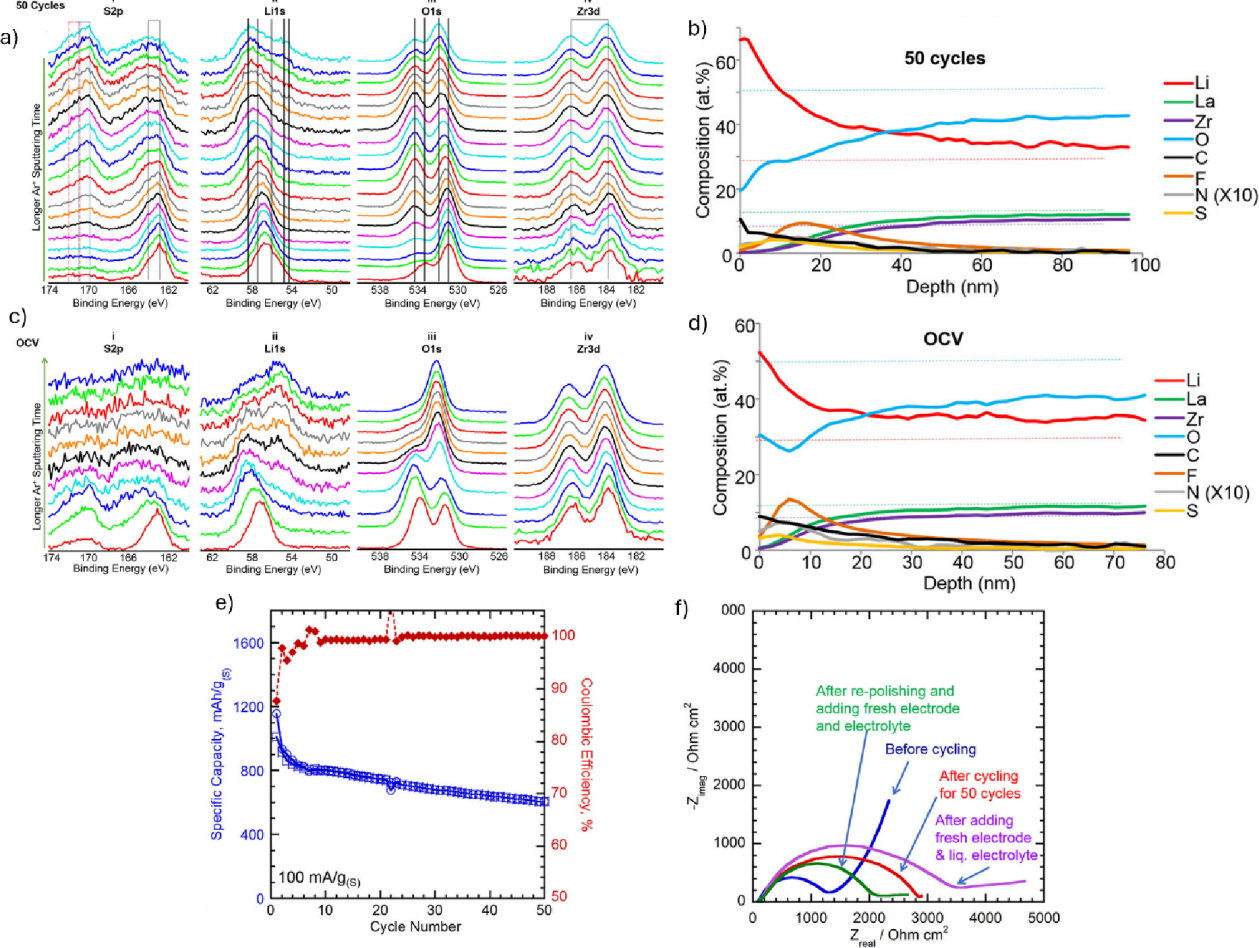
XPS depth spectra for LLZO from S/LLZO/Li hybrid cell (a) after 50 cycles and (c) after 18 h of open-circuit voltage. XPS depth profiles (b) after 50 cycles and (d) 18 h open-circuit voltage. (e) Cycling performance of the for S/LLZO/Li hybrid cell cycled between 1.5 and 3.0 V vs Li/Li+. (f) Niquist plot for S/LLZO/Li hybrid cell. Adapted from ref 56.

To tackle this issue, one of the proposed approaches is the deliberate replacement of certain ions with other cations that exhibit higher resistance toward sulfur. For instance, the aliovalent doping of LLZO with Ta^5+^ or Al^3+^ ions has shown promising results in terms of long-term durability.^57^ The cation replacement not only stabilizes the high-conductivity cubic phase of LLZO but also minimizes the formation of detrimental grain-boundary impurity phases that accelerate the degradation. As a result, this modification enhances both the chemical stability and the mechanical robustness of the material. However, further studies are still needed to completely understand the degradation process at the grain-boundary level and effectively tackle this issue. Theoretical calculations and modeling are expected to play a pivotal role in investigating the stability of oxides toward sulfides, as they are expected to unravel the phenomena occurring at the surface of oxides during the cycling, and it is still not clear. Understating in detail the phenomena occurring at grain boundaries will provide novel solutions, from the identification of elements with high resistance to polysulfide degradation to new oxide materials with superior performance that prevent the shuttle effect.^58^ Recent studies suggest that defective compounds can effectively adsorb polysulfide molecules, chemically blocking polysulfide migration. For instance, computational models suggest that oxygen vacancies in oxides, for example, in TiO_2_, can anchor within their surfaces polysulfides, preventing diffusion.^59^ Another approach considers the inclusion of thiophilic element oxides such as Mn_3_O_4_, thanks to the strong S–Mn interactions that link to polysulfides.^60^

Another approach considers the protection of oxide electrolytes through surface coating techniques to prevent direct contact with sulfur cathodes and block polysulfide diffusion. Materials like Al_2_O_3_, ZrO_2_, and LiNbO_3_ are commonly used as ceramic coatings due to their chemical inertness and rigidity that protect and block polysulfide diffusion.^61^ For instance, Al_2_O_3_ coatings can be applied using ALD to create uniform layers between the cathode and electrolyte surface, which shows a 57.8% higher discharge capacity compared to non-protected cells as shown by the study conducted by Meng *et al.* ^62^

Oxides exhibit strong resistance to polysulfide attack and diffusion, making them suitable for long-cycle lithium–sulfur batteries. However, degradation of the electrolytes, especially at the surface of the material, might lead to performance loss and polysulfide diffusion through grain boundaries and cracks in the material. Studies to improve stability are focused on advanced sintering techniques, which will synthesize more dense oxide-based electrolytes to minimize the presence of undesired grain boundaries. The exploration of advanced coating techniques and materials is also crucial for the ongoing development of more efficient and stable lithium–sulfur batteries that utilize oxide solid electrolytes.

Halide solid materials represent a promising and innovative class of inorganic electrolytes that are gaining attention in the field of energy storage solutions, yet studies for their use in Li–S batteries remain scarce. While these compounds demonstrate promising conductivities and elevated electrochemical stability when operating within the voltage range typical of Li–S batteries, they reveal significant challenges in terms of cycling performance to replace the preferred sulfide inorganic electrolytes. For instance, a research group led by Ohno et al. has investigated the chemical stability of three specific halide solid electrolytes in the presence of sulfur cathodes: Li_3_InCl_6_, Li_3_YCl_6_, and Li_3_YBr_6_.^63^ Basically, from this study, it was concluded that the halides show poor chemical stability with sulfur species. This incompatibility leads to the formation of M–S bonds (*M* = In or Y), since those metals prefer bonding with S instead of halogen elements, causing the degradation of the material.^64^ XPS analysis indicates the presence of S^2–^, which can be due to the presence of Li_2_S or LiYS_2_, yet the analysis performed is not able to accurately reveal the presence of Li_2_S or the metal halide due to the overlap of S 2p and Y 3d peaks. The reaction with sulfur manifests a significant obstacle in achieving reliable and efficient performance in battery applications, highlighting the need for further research and development in this emerging class of inorganic electrolytes. A potential solution could be the incorporation of oxygen into the inorganic halide electrolytes, which can protect the halides from sulfur by the formation of a dense ion-conductive interfacial layer. Zhang et al. demonstrated that introducing high concentrations of oxygen into a Zr-based solid electrolyte creates an oxy-halide phase with greatly improved compatibility toward sulfide electrolytes. This dual-anion engineering lowers interfacial reaction energies and promotes a dense ion conductive Li_3_PO_4_-rich layer at the halide–sulfide interface, showing stable cycling beyond 400 cycles.^65^ This research offers important understanding and approaches to enhance the compatibility of halides and sulfides, promoting the progress of all-solid-state batteries.

Protective coatings and interlayers have been proposed to mitigate the instability of halide solid electrolytes. For example, polymer coatings such as PMMA on Li_3_YCl_6_ particles have shown to suppress side reactions and improve interface durability in solid-state cells by preventing direct electrolyte degradation.^66^ Recent studies have explored the direct incorporation of halide solid electrolytes into sulfur cathodes as a strategy to enhance the interfacial stability and cycling performance in all-solid-state Li–S batteries. Fang et al. demonstrated that introducing a halide phase as a catholyte within the composite sulfur cathode improves lithium transport, maintains interfacial integrity, and suppresses capacity decay during cycling.^67^ Unlike traditional approaches, inclusion within the cathode provides a chemically stable, high-oxidation stability interface that mitigates polysulfide-induced degradation. This approach illustrates that halide electrolytes can play a dual role: serving both as ion conductors and as protective interfacial agents within the cathode, complementing strategies such as polymer coatings or buffer layers to preserve the integrity of the halide–sulfur interface.

In conclusion, inorganic halides are still in early research stages of development, with promising applications in all solid-state batteries; however, more studies are needed to incorporate these electrolytes into lithium–sulfur batteries effectively. Some promising approaches can include protection from direct contact with sulfur cathodes, given their poor chemical stability, such as coatings or the fabrication of hybrid materials with polymeric materials to avoid degradation in the presence of polysulfides. More recent solutions explored the use of halide electrolytes as cathodes within the sulfur cathode, which opens the door to designing advanced sulfur cathodes with enhanced resistance toward polysulfide-induced degradation and improving long-term performance of all-solid-state Li–S batteries.

## 3 Polysulfide Diffusion in Polymer Electrolytes

Polymer electrolytes are used in lithium–sulfur (Li-S) batteries by combining a polymer matrix with a lithium salt. The most common polymers are poly­(ethylene oxide) (PEO) and poly­(vinylidene fluoride) (PVDF). From the seminal work from Marmorstein *et al.*,^68^ it becomes evident that polysulfide shuttling does occur in ethylene oxide (EO)-based SPEs. Moreover, Balsara and co-workers developed an XAS-based calibration method, based on first-principles calculations, to probe the polysulfide speciation in EO-based SPEs.^69,26^ The studies reveal that despite the challenge related to the reversible disproportionation of the polysulfide intermediates, the XAS fingerprint of polysulfide species can be elucidated by the linear dependency between the ratio of the two main absorption features derived from terminal- and inter-chain sulfur atom signals near the sulfur K-edge.^26^ Based on the XAS fingerprint established, Balsara and co-workers were able to probe the polysulfide diffusion into an EO-based SPE, near the anode electrode at different discharge stages (i.e., 2.25, 2.02, and 1.50 V vs Li/Li^+^) with a subsequent 3-day equilibrium rest at 90 °C.^70^ It is noteworthy that polysulfide species detected can be generated by either electrochemical reactions or subsequent chemical disproportionation reactions. The study revealed that polysulfide radical anions (i.e., LiS_5_, LiS_4_, and LiS_3_) and long-chain polysulfide dianions (i.e., Li_2_S_6_ and Li_2_S_8_) are dominantly present in the EO-based SPE after the first discharge stage (i.e., 2.25 V vs Li/Li^+^). It is noteworthy that the absence of short-chain polysulfide dianions (i.e., Li_2_S_*x*_, *x* = 2–5) indicates a rather long-term stability of the long chain polysulfide dianions. After the second discharge stage (i.e., 2.02 V vs Li/Li^+^), the polysulfide composition in the EO-based SPE is dominated by short-chain polysulfides and Li_2_S, with still some long-chain polysulfide dianions still detected. At the end of the discharge, long-chain polysulfide dianions are no longer detected and the EO-based SPE composition is dominated by short-chain polysulfides and Li_2_S. Using a similar approach, Balsara and co-workers also provided insight into the reasons behind the low resulting capacity of thick sulfur solid-state polymer cathode (i.e., 15 % in this case, compared to theoretical value), probing that 58% of elemental sulfur at the back of the cathode is really consumed by a combination of both electrochemical and chemical disproportionation reactions due to both limited lithium-ion diffusion and polysulfide diffusion from the front to the back of the cathode.^71^ Miller *et al.* reported the use of X-ray absorption spectromicroscopy to probe sulfur reaction mechanisms and polysulfide diffusion via *operando* spatially resolved chemical mapping of a cross-sectional Li–S battery, based on an EO-based SPE.^72^ The study revealed that only a small extent of the S_8_ is recovered after the first charge with mainly long-chain polysulfide dianions (i.e., Li_2_S_8_) detected in the cathode. It is also noteworthy that Li_2_S is still detected after the third charging process, highlighting again some irreversibility in the sulfur reactions.

Marceau et al. reported the use of operando UV-visible spectroscopy to probe the accumulation of polysulfide species in a crosslinked EO-based SPEs.^73^ Similar to XAS studies, the obtention of UV-visible fingerprints of distinguished polysulfide species is challenging. In this case, the authors proposed a band assignment method based on polysulfide solution in THF using a combination of UV-visible and HPLC techniques. Based on the UV-visible band assignment obtained, the study reveals the accumulation of short-chain polysulfide (i.e., S_4_^2–^) into the SPE during the discharge process, while an accumulation of long-chain polysulfide (i.e., S_6_^2–^) was observed during the charge process.

In an *ex situ* Li–S battery approach, Balsara and co-workers reported the use of electrochemical impedance spectroscopy to characterize the morphology, thermal, and transport properties (i.e., total ionic conductivity) of polysulfide species (i.e., Li_2_S_4_ and Li_2_S_8_) dissolved in an EO-based polymer matrix (i.e., PEO or PS–PEO (SEO)).^74^ The study showed that the Li_2_S_8_/EO-based polymer interactions are more favorable than that for the Li_2_S_4_/EO-based polymer, as suggested by the more pronounced reduction of the crystallinity degree of EO chains. In terms of transport properties, the polysulfide species/EO-based polymer showed lower total ionic conductivity when compared to the LiTFSI/EO-based polymer, with the following ionic conductivity trend: LiTFSI/EO-based polymer > Li_2_S_8_/EO-based polymer > Li_2_S_4_/EO-based polymer. In a similar *ex situ* Li–S battery approach, Ahiavi *et al.* reported the lithium transference number and ambipolar diffusion coefficient of Li_2_S_*x*_/EO-based polymer systems, using the Bruce and Vincent method and restricted diffusion technique from Newman and Chapman, respectively.^75,76^ The studies revealed that the Li_2_S_*x*_/EO-based polymer systems displayed a higher lithium transference number when compared to the LiTFSI/EO-based polymer one, suggesting that the S_*x*_^–^ anion has more mobility than that of the TFSI anion. These results agreed with the calculated ambipolar diffusion coefficients, highlighting that the LiTFSI/EO-based polymer and the Li_2_S_*x*_/EO-based polymer system showed similar lithium diffusion coefficients, while the S_*x*_^–^ diffusion coefficients are almost one order of magnitude lower than that of TFSI^–^.

Unlike polymers with a high donor number, such as PEO, which facilitate the generation of soluble polysulfides, PVDF exhibits a low donor number.^73^ This property renders polysulfides insoluble and unstable within the PVDF matrix, thereby promoting a direct solid–solid conversion of sulfur into Li_2_S_2_/Li_2_S without the formation of soluble intermediates, as evidenced by the single-discharge plateau observed in the voltage profile, in contrast to the multiple plateaus typical of reactions involving soluble intermediates.^73^ It should be noted, however, that PVDF shows weak affinity and limited adsorption capacity toward polysulfides,^69^ suggesting that its primary role lies in preventing polysulfide generation rather than actively adsorbing them once formed. For example, Fang *et al.* reported the use of PVDF/LiTFSI and PEO/LITFSI as cathode binder and SPE, respectively, resulting in improved cycling stability.^77^ This approach plays on the insolubility of long-chain polysulfide in the cathode binder to trigger a change in the sulfur redox reaction mechanism from a multistep “solid-liquid-solid” reaction to a single step “solid–solid” reaction. In summary, polymer electrolytes with a low donor number are the most promising ones for Li–S batteries, and further development should focus on novel polymeric materials that can prevent polysulfide shuttle.

## 4 Polysulfide Diffusion in Hybrid Electrolytes

Hybrid solid electrolytes (HSEs) represent a promising strategy as they are designed to combine the advantages of solid polymer electrolytes (SPEs) such as flexibility, good interfacial contact, and ease of processing, with the superior properties of inorganic solid electrolytes (ISEs), including high ionic conductivity, mechanical strength, and chemical or thermal stability.^78–89^ Moreover, they might overcome the critical challenges faced by lithium–sulfur (Li–S) batteries, particularly the “polysulfide shuttle effect” ^85–88,89^ and the growth of lithium dendrites.^78,83,90–92^

In the context of HSEs, inorganic fillers can be incorporated into the polymer matrix to optimize its structural, mechanical, and electrochemical properties. These fillers are generally classified into two main categories: passive (inert) fillers and active (ion-conducting) fillers.^93^ Passive fillers, although not directly contributing to Li^+^ transport, exert significant influence through structural and interfacial modifications of the polymer matrix. By reducing polymer crystallinity, they promote the formation of amorphous domains, which lower the activation energy barriers and improve ion mobility.^92,94–102^ In addition, their surface functional groups interact with lithium salt anions, weakening Li^+^–anion coordination, enhancing salt dissociation, and indirectly improving ionic conductivity.^81,88,94,98,103–105^ From a mechanical standpoint, passive fillers reinforce the polymer matrix, providing higher elastic modulus to suppress lithium dendrite growth.^81^ Furthermore, the addition of inert filler can improve interfacial compatibility between the electrolyte and electrodes, which leads to stabilizing the formation of the solid electrolyte interphase (SEI).^79,80,94,106–110^ A well-formed SEI ensures homogeneous Li^+^ transport, suppressing dendrite formation and reducing interfacial resistance.^79,80^ Common examples include metal oxides such as Al_2_O_3_,^80,106,111,112^ SiO_2_,^81,99,102,110,113,114^ In_2_O_3_,^103^ ZrO_2_,^102,109^ or TiO_2_,^101,102,111,115,116^ as well as carbon-based materials; layered silicates such as vermiculite,^105^ halloysite nanotubes (HNTs),^117^ montmorillonite (MMT),^118^ multilayered structures,^79,80,111,119–121^ and sandwich-type composites;^122,123^ and more complex structures like metal-organic frameworks (MOFs)^94,104^ and covalent organic frameworks (COFs).^96^

Passive inorganic fillers can interact through dipole–dipole interactions^103,106^ and chemical adsorption^115^ with the polysulfide species, reducing or avoiding the shuttle mechanism. In physical confinement, the solid nature of the electrolyte prevents the free diffusion of polysulfides.^79,88,103,118,119,121,122,124–127^ The incorporation of inert inorganic fillers, such as Al_2_O_3_, SiO_2_, In_2_O_3_, ZrO_2_ **,** or TiO_2_, into the polymer matrix disrupts its crystallinity, promoting the formation of amorphous regions^81,102,110–112^ that enhance lithium-ion transport.^101,102,109–112,116^ Simultaneously, these fillers physically confine polysulfides by introducing structural barriers that inhibit their dissolution and limit their migration.^80,106,109,112,114^ Judez et al.^106^ investigated the role of aluminum oxide (Al_2_O_3_) as both an electrolyte filler and a cathode additive in solid-state Li–S batteries, emphasizing its ability to adsorb lithium polysulfides. Al_2_O_3_ can effectively trap polysulfides within the cathode through reversible adsorption, thereby preventing the loss of active material. Visual experiments, in which Al_2_O_3_ nanoparticles were added to a Li_2_S_6_ solution in 1,2-dimethoxyethane, revealed a color change from brownish-green to colorless, directly evidencing the strong interaction between ceramic particles and polysulfides. However, when added only to the electrolyte, Al_2_O_3_ may induce capacity loss if adsorbed polysulfides lose electrical contact with conductive materials. To overcome this, an optimized configuration is proposed in which Al_2_O_3_ is also introduced into the cathode, acting as a polysulfide reservoir. The optimized configuration enabled a capacity of 0.85 mAh cm^–2^ and 755 mAh g^–1^ at C/10 after 30 cycles with high coulombic efficiency (Figure 3). Jeong *et al*.^112^ explored a PEO-based electrolyte containing 10 wt % Al_2_O_3_ and LiBF_4_. The addition of Al_2_O_3_ contributed to polysulfide immobilization through strong adsorption while also stabilizing Li_2_S formation and improving sulfur utilization. Lee *et al*.^81^ introduced an HSE containing highly porous rice husk-derived silica (RHSiO_2_). The dissolution of sulfur was effectively inhibited, and no polysulfide infiltration was observed in the HSE after 100 cycles, indicating an improved long-term battery stability. Zhang *et al.* ^103^ researched an HSE by adding nanoparticles of In_2_O_3_ to a PEO matrix. In_2_O_3_ nanoparticles effectively trap polysulfides, thereby suppressing the shuttle effect, as confirmed by H-type permeation experiments, where the PEO/LiTFSI/In_2_O_3_ electrolyte successfully inhibited spontaneous polysulfide diffusion for over 12 h, unlike the In_2_O_3_ free system. XPS analysis of the lithium anode surface after cycling further demonstrated the adsorption of polysulfides by In_2_O_3_, showing reduced intensity and fewer polysulfide species compared to those of the control electrolyte, with no evidence of Li_2_S_2_ formation. Moreover, the interaction of In_2_O_3_ with TFSI^–^ anions, particularly with −CF_3_ and −SO_2_– groups, promoted lithium salt dissociation and released additional Li^+^, thereby enhancing the ionic conductivity through a clear chemical interaction mechanism.

**Figure 3:**
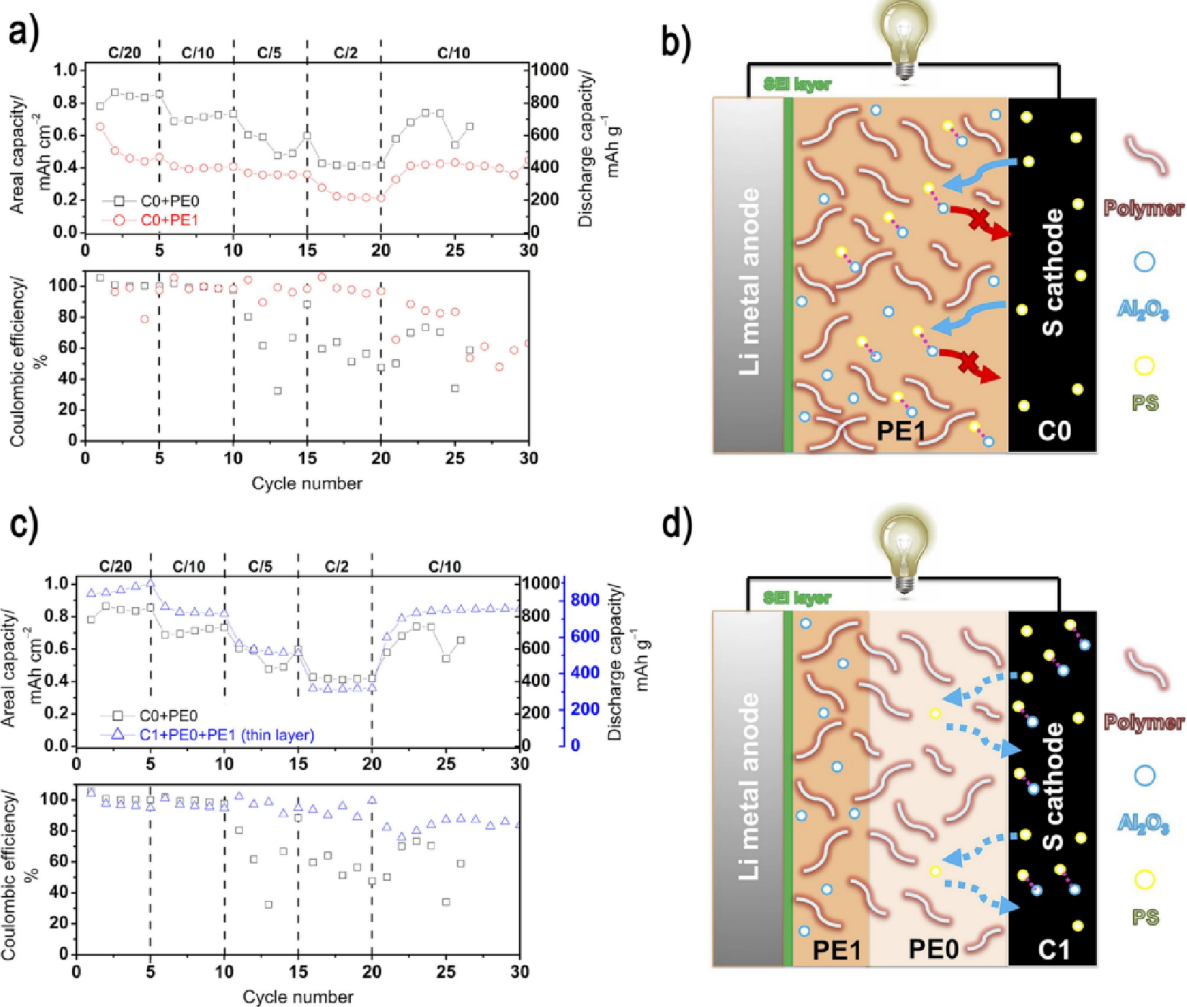
Cycling performance at different discharge/charge rates of (a) filler containing electrolyte cell, C0 + PE1, compared to the reference filler free C0 + PE0 cell, along with (b) the schematic representation of the C0 + PE1 system. Cycling performance of (c) optimized C1 + PE0 + PE1 system, compared to the reference filler free C0 + PE0 cell, along with (d) the schematic representation of C1 + PE0 (dark blue) + PE1 (light blue) system. Reproduced with permission of ref 106.

Advanced materials such as vermiculite,^105^ halloysite nanotubes (HNTs),^117^ montmorillonite (MMT),^118^ multilayered structures,^79,80,111,119–121^ and sandwich-type composites,^122,123^ which combine “polymer-in-ceramic” and “ceramic-in-polymer” layers, offer specialized functions, including enhanced lithium-ion transport, dendrite suppression, and polysulfide blocking due to physical confinement effects. Zhai *et al*.^105^ proposed a laminar composite solid electrolyte (LCSE), designated Vr/PEO-LCSE, synthesized by filtration of vermiculite nanosheets followed by the intercalation of PEO–LiTFSI through a swelling/filtration method. The layered stacking of large vermiculite nanosheets effectively suppressed polysulfide diffusion, endowing the electrolyte with a strong polysulfide blocking capacity, high mechanical robustness, and a favorable lithium transference number (t^+^), which together contributed to the superior cycling stability of Li|Vr/PEO-LCSE|S cells. The minimal variation in charge transfer resistance during cycling corroborated the LCSE’s efficiency in inhibiting polysulfide migration compared with other electrolyte systems. Zhang *et al*.^118^ studied a hybrid solid electrolyte based on PEO, LiTFSI, and montmorillonite (MMT) nanoclay. The solid-state configuration of the PEO/LiTFSI/MMT electrolyte functioned as a physical barrier that mitigated polysulfide dissolution from the sulfur cathode and protected the lithium anode, making it a promising candidate for stable all-solid-state Li–S batteries. Elizalde-Segovia *et al*.^79^ describe a bilayer solid electrolyte configuration. The electrolyte consists of a mixed conduction membrane (MCM) made of lithiated cobalt oxide (LCO, ∼30 μm thick), and a polymer electrolyte layer composed of PEO with LiTFSI at a molar ratio of 8:1 (10–30 μm thick), in direct contact with the lithium anode. To enhance ionic conductivity and mechanical robustness, alumina (Al_2_O_3_) nanoparticles (10 wt % relative to PEO and LiTFSI) were incorporated into the polymer layer. This bilayer electrolyte effectively suppresses polysulfide dissolution and shuttle effects by retaining sulfur species in the solid state. The electrolyte with alumina retains 60% of initial capacity after 8 cycles in a C/20, while the electrolyte without alumina doping drops to 15%. The study relates the capacity loss due to cathode degradation, mainly sulfur volume expansion, leading to an increase of charge transfer resistance. As a result, the Li–S cell with the proposed hybrid demonstrates a high initial specific capacity of up to 1481 mAh g^–1^ at C/20 (88% of the theoretical value with Al_2_O_3_) and low internal resistance comparable to liquid-based Li–S cells.

In Li–S batteries, electrolytes incorporating metal-organic frameworks (MOFs)^94,104^ and covalent organic frameworks (COFs)^96^ have been widely employed to mitigate the polysulfide shuttle effect. Both MOFs and COFs are porous crystalline materials with unique structural features that enable them to act as physical barriers or chemical mediators for polysulfides while simultaneously enhancing lithium-ion transport. Suriyakumar *et al*.^94^ studied an HSE based on a PEO network containing LiTFSI and an aluminum terephthalate MOF (Al-TPA-MOF). The incorporation of Al-TPA-MOF into the PEO-based composite electrolyte provided multiple functions that contributed to polysulfide regulation in Li–S batteries. Specifically, Al-TPA-MOF acted as a scavenger by removing residual solvent, water, and other impurities, thereby preventing their accumulation at the lithium/HSE interface, which enhanced interfacial stability and delayed polysulfide migration. In addition, the Lewis acidic centers of Al-TPA-MOF can interact with the lithium salt anion, leading to a reduced crystallinity of the host polymer and promoting salt dissociation, effects that indirectly influence polysulfide behavior. X-ray photoelectron spectroscopy (XPS) performed after cycling further confirmed the suppression of lower-order polysulfide transport across the HSE, even after 50 cycles, although a low-intensity signal of higher order polysulfides was still detected, highlighting the need for further mechanistic investigations (Figure 4).

**Figure 4:**
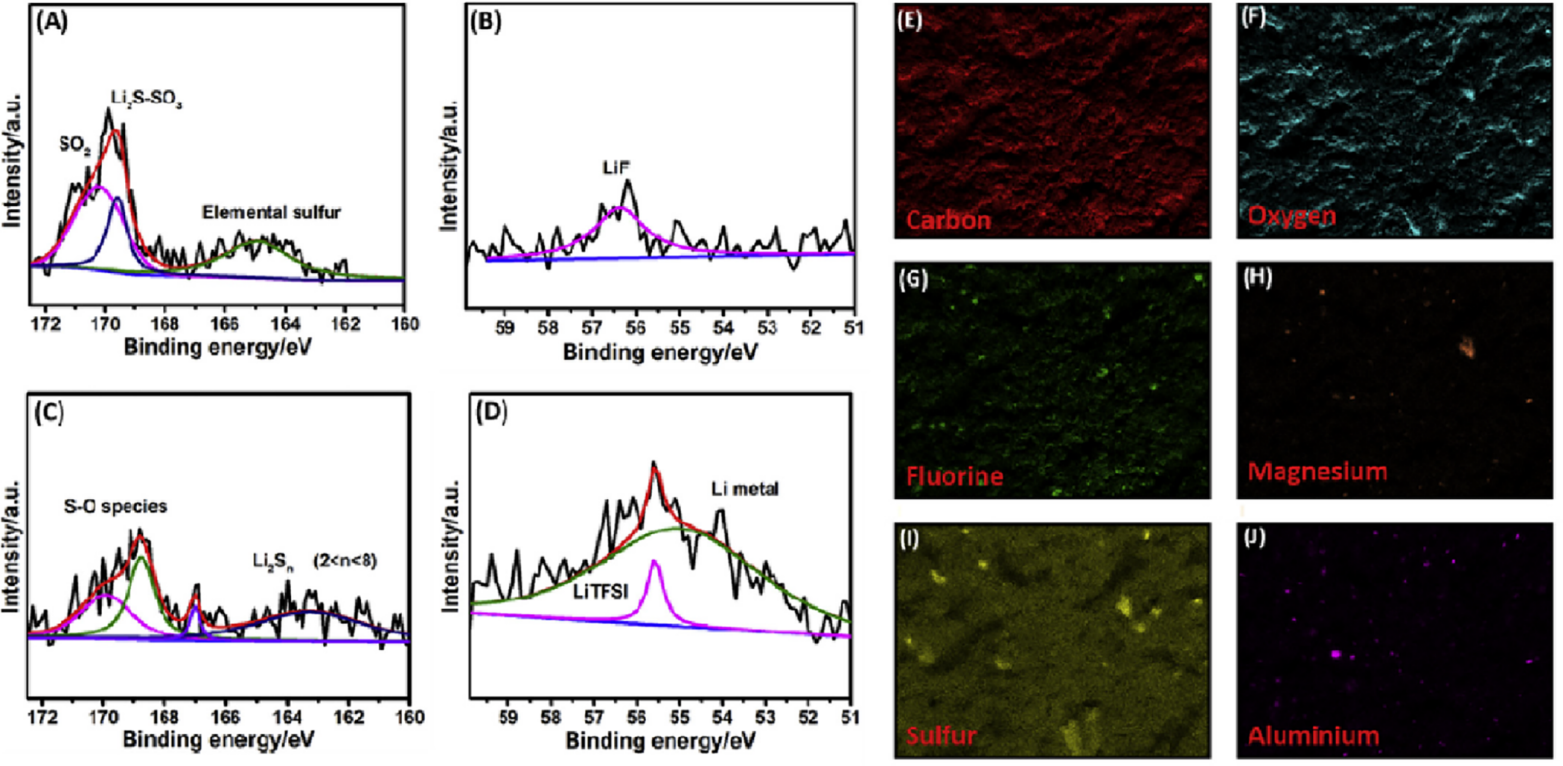
Post-cycling XPS analysis of the S4 CPE: (a) S_2p_, (b) Li_1s_ on the cathode side, and (c) S_2p_, (d) Li_1s_ on the anode side, along with the following elemental mappings of (e) carbon, (f) oxygen, (g) fluorine, (h) magnesium, (i) sulfur, and (j) aluminum. Reproduced with permission of ref 94 Copyright 2018, Elsevier.

Li *et al*.^104^ propose a PEO-based electrolyte with ordered MIL-125–NH_2_ as a filler. The HSE was prepared by electrospinning, followed by calendaring, and then infiltrated with a homogeneous PEO/LiTFSI solution. The ordered MIL-125–NH_2_ structure provided multiple barriers to prevent polysulfide shuttling. The highly ordered micropores (∼4.5 Å) of MIL-125–NH_2_ were smaller than the diameters of polysulfides (Li_2_S_n_, 4 < *n* ≤ 8, 5.2–8.4 Å), physically restricting them. The open Ti sites in the ordered MOF structure interact more strongly with TFSI^–^ ions, facilitating LiTFSI dissociation, increasing Li^+^ concentration, and consequently enhancing ionic conductivity and the Li^+^ transference number. The compact, ordered 3D network structure of MIL-125–NH_2_ provided continuous pathways for Li^+^ conduction and chemically interacted with anions, limiting their movement and suppressing polysulfide shuttle (Figure 5). *In situ* Raman tests and lithium polysulfide permeation experiments confirmed that the 3D MPPL CSE electrolyte efficiently suppresses the shuttling compared with electrolytes without MOF or with disordered MOF.

**Figure 5:**
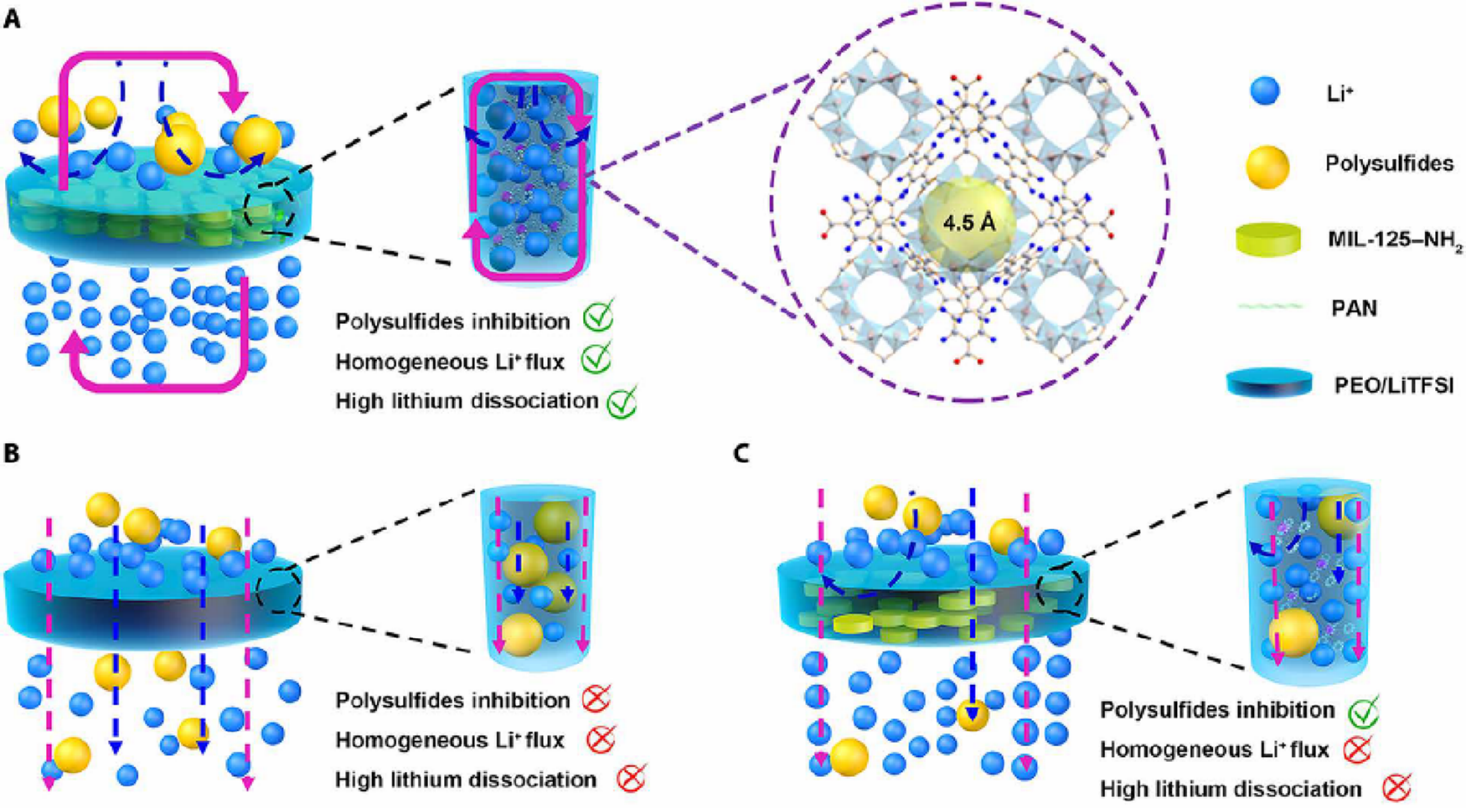
Schematic images of three CSEs in the application of ASSLSBs. (A) 3D MPPL CSE, (B) PL CSE, and (C) MPL CSE. Reproduced with permission of ref 104 Copyright 2024, AAAS.

In a study, Wang *et al*.^96^ proposed an electrolyte that incorporates nanosheets of a zwitterionic COF linked by squaraine, called HUT4, in a PEO matrix that also contain LiTFSI. Zwitterionic HUT4 exhibited strong adsorption of lithium polysulfides (Li_2_S_6_). Experiments showed that a Li_2_S_6_ solution with HUT4 displayed weaker residual Li_2_S_6_ signals compared to the original Li_2_S_6_ solution and pure PEO solution, indicating strong adsorption. The zwitterionic structure of HUT4 is key for polysulfide blocking, providing abundant active sites to capture polysulfide through strong interactions. Positively charged N atoms in HUT4 interacted with Sx^2–^ anions (from polysulfides) while negatively charged O atoms in HUT4 interacted with Li^+^ cations. This dual strong interaction allowed HUT4 to effectively capture lithium polysulfides, suppressing the shuttle effect and improving battery performance.

Active fillers directly participate in Li^+^ transport due to their intrinsic ionic conductivity and high lithium-ion transference numbers.^89,97^ By integrating ion-conducting phases into the polymer matrix, they provide additional diffusion channels for Li^+^ beyond those offered by the polymer chains.^92,128^ At suitable concentrations, these fillers can even form continuous ion-conduction networks, as observed in nanofibrous^92^ or three-dimensional architectures.^129^ Active fillers not only enhance ionic transport but also contribute to mechanical reinforcement^82,85,93,97,100,113,128–130^ and act as a barrier against lithium polysulfides.^131^ Through adsorption and retention mechanisms, they inhibit dendrite growth, mitigate the shuttle effect, and regulate polysulfide redox chemistry by restricting their migration to the anode while enabling controlled utilization at the cathode. Representative active fillers include garnet-type oxides such as Li_7_La_3_Zr_2_O_12_ (LLZO),^95,128,129^ Li_6.5_ La_3_Zr_1.5_Ta_0.5_O_12_ (LLZTO),^82,121,130^ Li_6.28_ La_3_Al_0.24_ Zr_2_O_12_ (LLAZO),^78^ and Li_6.4_La_3_B_0.5_Zr_1.4_Ta_0.6_O_12_ (B = Al, Ga, Nb, etc.) (LLBZTO)^90^ and NASICON-type materials such as Li_1.3_Al_0.3_Ti_1.7_(PO_4_)_3_ (LATP)^125^ and Li_1.5_Al_0.5_Ge_1.5_(PO_4_)_3_(LAGP).^83,132^ Another family of active fillers is the lithium sulfide-based conductors like Thio-LISICON sulfides such as Li_10_SnP_2_S_12_ (LSPS)^97,131^ and Li_10_GeP_2_S_12_ (LGPS),^85^ sulfide glass-ceramic such as Li_3_PS_4_ ^84^ and Li_7_P_3_S_11_,^133^ and argyrodite sulfides such as Li_6_PS_5_ *X* (*X* = Br, Cl, I).^93^ A further group comprises other lithium-ion conducting oxides such as perovskite oxides as Li_3*x*_La_2/3–*x*_TiO_3_, where 0 < *x* < 0.16 (LLTO)^92^ and LiAlO_2_.^100^ Finally, halides such as Li_3_InCl_6_ ^134,135^ are also present in hybrid solid electrolytes. However, it is worth noting that garnet-type oxides are the most promising active fillers. Cheng *et al*.^78^ developed a hybrid solid electrolyte by incorporating electrospun and calcinated LLAZO nanofibers into a PEO-based polymer matrix, forming a well interconnected organic–inorganic 3D structure that facilitates continuous Li^+^ conduction. The garnet-type LLAZO nanofibers have strong affinity for polysulfides, which are effectively blocked via two mechanisms: a physical barrier provided by the mechanically reinforced nanofibers and Lewis acid–base interactions between LLAZO and polysulfide anions that restrict their mobility. Post-cycling analysis confirmed reduced polysulfide deposition on lithium anodes compared to that on a pure polymer electrolyte. The hybrid electrolyte enabled stable cycling over 100 cycles with an initial discharge capacity of 968 mAh g^–1^ and 61% capacity retention, as well as high-rate capability, highlighting its potential as a robust, high-performance electrolyte for Li–S batteries.

In addition to the inorganic phase, the organic matrix plays a crucial role in the structure and properties of an HSE. The vast majority of studies on hybrid solid electrolytes for Li–S batteries have focused on PEO or PEO-based systems, due to its high Li^+^ solvation ability, flexibility, electrochemical stability, and mature technological development.^98^ However, studies have showed that polysulfides are highly soluble in PEO^82,73^ as we described in the previous section. To allow the utilization of PEO-based HSEs in Li–S batteries, one strategy is relying on the efficiency of the previously discussed inorganic materials to mitigate and/or block the polysulfide migration. Nonetheless, the substitution of PEO by other polymers might be a more effective solution. Hybrid systems incorporating alternative polymers, such as PMMA (poly­(methyl methacrylate)^108,136,137^ or PVDF (poly­(vinylidene fluoride)),^107,73^ have also been investigated for Li–S batteries; however, in these cases, a liquid solvent is usually introduced to enhance ionic transport within the polymer matrix. This addition, in practice, transforms the material to a hybrid/composite gel electrolyte. Another alternative strategy is a blend of polymers. For example, Mashekova *et al*.^138^ used spin-coating to produce a S/CNT/Super P cathode and a composite electrolyte based on PEO, PVDF, LiTFSI, and LLZO. The quasi-solid composite electrolyte (PEO/PVDF/LiTFSI/LLZO) was designed to suppress polysulfide dissolution, enhance ionic conductivity, and stabilize the electrode–electrolyte interface. The optimal composition ratio of PEO/PVDF/LiTFSI was 7:3:2, with LLZO incorporated up to 25 wt %, significantly reducing both bulk and interfacial resistance, effectively suppressing the polysulfide shuttle effect and enhancing the structural stability and ionic conductivity of the Li–S cell. In addition, the use of computational modeling based on density functional theory (DFT) assists in predicting molecular interactions between electrolytes and polysulfides, enabling more efficient rational design.^85,87,115,139–142^ Lee *et al*.^115^ demonstrated that the undesired transport of dissolved polysulfides, known as the shuttle effect, can be effectively suppressed by incorporating TiO_2_ nanoparticles into a solid PEO-based nanocomposite electrolyte. Raman spectroscopy performed on the lithium anode after 10 cycles revealed that in the PEO/TiO_2_ system, no signatures of polysulfides or Li_2_S were present, in contrast to the pure PEO electrolyte. Additional Raman peaks attributed to polysulfides were observed only on the cathode-facing side of the PEO/TiO_2_ electrolyte, indicating that TiO_2_ nanoparticles effectively trap polysulfides and inhibit their migration toward the anode. DFT calculations supported these findings, showing that the adsorption energy of Li_2_S_*n*_ and sulfur species on the anatase TiO_2_ (101) surface is an order of magnitude higher than that on PEO or graphite. The mechanism involves strong binding of lithium ions from Li_2_S_*n*_ to surface oxygen atoms of TiO_2_, weaker adsorption of polysulfide anions on surface Ti, and electron transfer from polysulfides to Ti sites, resulting in ionic bond formation and efficient suppression of polysulfide diffusion. The high surface area of TiO_2_ nanoparticles further enhances polysulfide binding, thereby minimizing the shuttle effect.

In hybrid electrolytes, PVDF and its copolymers, such as poly­(vinylidene fluoride-*co*-hexafluoropropylene) (PVDF-HFP), are well known for suppressing the formation and dissolution of long-chain lithium polysulfides.^99,133,135,137^ PMMA, particularly due to its ester (C=O) functional groups, has demonstrated a strong ability to trap or chemically anchor dissolved lithium polysulfides.^136,137^ Similarly, poly­(m-phenylene isophthalamide) (PMIA)^143^ and polyacrylonitrile (PAN)^139^ have shown promising roles when incorporated into HSEs via electrospinning; in PMIA, −NH_2_ groups from components such as octa­(aminophenyl)­silsesquioxane (OAPS) effectively confine polysulfides,^143^ while in PEO-based copolymers, the formation of C=N–O functional groups during crosslinking with PAN efficiently suppresses polysulfide shuttling.^139^ For applications beyond Li–S batteries, HSEs with a more diverse selection of polymers are reported, from conducting polymers^141,144^ to non-conducting polymers.^134,135^

Alternatively, single-ion conducting polymers could help mitigate polysulfide shuttling.^125,126,145^ However, the use of hybrid single ion-conducting electrolytes in solid-state Li–S batteries to avoid polysulfide shuttle has not been widely reported in the literature. Villaluenga *et al*.^126^ developed a non-flammable hybrid solid electrolyte by covalently linking sulfide glass particles (75Li_2_S·25P_2_S_5_) with a perfluoropolyether (PFPE) polymer matrix. X-ray absorption spectroscopy (XAS) confirmed that the hybrid electrolyte effectively suppressed lithium polysulfide dissolution by excluding Li_2_S_8_ from its structure. As a result, the hybrid electrolyte efficiently mitigated the shuttle effect. Therefore, the use of hybrid single ion-conducting electrolytes as a solution for polysulfide shuttle, although promising, remains largely unexplored.

In summary, hybrid solid electrolytes (HSEs) represent a strong contender for overcoming polysulfide shuttle in Li–S batteries, as their solid nature and the use of inert or active fillers help to adsorb, entrap, and block polysulfide migration, while designing strategies like multilayer structures, crosslinking, and fabrication methods like electrospinning increase the stability and durability of the system. Nevertheless, the challenge in HSEs resides in optimizing them in a way that the blocking mechanism is not compromised by the residual solubility of the polymeric matrix or by adverse effects related to the inorganic phase.

## 5 Summary and Future Directions

Solid-state electrolytes offer a compelling alternative to traditional liquid electrolytes by providing a stable framework that can potentially suppress polysulfide dissolution and migration in Li–S batteries. The chemical composition of solid electrolytes affects the polysulfide solubility. Solid electrolytes based on inorganic materials, such as sulfide- and halide-based material protective coatings, are needed to mitigate interfacial issues. Oxide-based solid electrolytes are less prone to react with polysulfides compared with their sulfide or halide counterparts, making them attractive for Li–S battery applications. However, polysulfide diffusion through grain boundaries remains a challenge, particularly during extended cycling. Advanced sintering techniques, such as spark plasma sintering and microwave sintering, have been employed to produce dense, pore-free oxide electrolytes that minimize polysulfide penetration. Polymer electrolytes, particularly those based on poly­(ethylene oxide) (PEO), present high solubility of polysulfides in PEO-based matrices and exacerbate the shuttle effect, leading to capacity fading and reduced cycling stability. To address this issue, researchers have explored alternative polymers, such as poly­(vinylidene fluoride) (PVDF) and poly­(methyl methacrylate) (PMMA), which exhibit lower polysulfide solubility. An ideal approach would therefore be to design polymer electrolyte matrices intrinsically insoluble in polysulfides, addressing the shuttle problem at its origin in polymer electrolytes. The development of single-ion conducting electrolytes, along with a deeper understanding of adsorption and catalytic mechanisms, is also essential to develop efficient polymer-based electrolytes and even hybrid electrolytes. Hybrid solid electrolytes represent a promising class of materials that combine the advantages of inorganic fillers (inert or active) and polymer matrices, offering a synergistic approach to suppress polysulfide migration while maintaining mechanical flexibility and ionic conductivity. The current most promising materials are a blend of non-conducting (non-polar) polymers and active inorganic fillers (preferably, garnet-type oxides) that synergistically should prevent polysulfide shuttle, leading to high-performance Li–S batteries. Overall, future research on polysulfide mitigation could aim to integrate physical, chemical, and catalytic strategies in a synergistic manner, regardless of the solid-state electrolyte. Moreover, computational modeling, including density functional theory (DFT), is expected to play a crucial role in predicting the interactions between polysulfides and polymer or inorganic matrices, which would enable the rational design of more efficient solid-state electrolytes for Li–S battery applications.
